# Extracellular dGMP Enhances *Deinococcus radiodurans* Tolerance to Oxidative Stress

**DOI:** 10.1371/journal.pone.0054420

**Published:** 2013-01-24

**Authors:** Mingfeng Li, Hongxing Sun, Qiong Feng, Huiming Lu, Ye Zhao, Hui Zhang, Xin Xu, Jiandong Jiao, Liangyan Wang, Yuejin Hua

**Affiliations:** 1 Key Laboratory for Nuclear-Agricultural Sciences of Chinese Ministry of Agriculture and Zhejiang Province, Institute of Nuclear-Agricultural Sciences, Zhejiang University, Hangzhou, China; 2 Key Laboratory of Stem Cell Biology, Institute of Health Sciences, Shanghai Institutes for Biological Sciences, Chinese Academy of Sciences and Shanghai Jiao Tong University School of Medicine, Shanghai, China; 3 Shanghai Institute of Immunology, Institutes of Medical Sciences, Shanghai Jiao Tong University School of Medicine, Shanghai, China; Louisiana State University and A & M College, United States of America

## Abstract

Free extracellular DNA provides nutrition to bacteria and promotes bacterial evolution by inducing excessive mutagenesis of the genome. To understand the influence of extracellular DNA fragments on *D. radiodurans*, we investigated cell growth and survival after extracellular DNA or dNMPs treatment. The results showed that the extracellular DNA fragments inhibited the growth of *D. radiodurans*. Interestingly, dGMP, a DNA component, enhanced *D. radiodurans* tolerance to H_2_O_2_ and gamma-radiation significantly. Further experiments indicated that extracellular dGMP stimulated the activity of one catalase (KatA, DR1998), and induced gene transcription including *the extracellular nuclease* (*drb0067*). When this only extracellular nuclease gene (*drb0067)* in *D. radiodurans* was deleted, the mutant strain showed more sensitive to H_2_O_2_ and gamma-radiation than the wild type strain. These results suggest that DRB0067 plays an important role in oxidative stress resistance. Taken together, we proposed a new anti-oxidation mechanism in *D. radiodurans*. This mechanism acts to increase expression levels of DRB0067 which then secretes active nuclease to degrade extracellular DNA fragments. The extracellular nuclease has a two-fold benefit, creating more free dNTPs for further cell protection and the removal of extracellular DNA fragments.

## Introduction

Bacteria cell death releases cytoplasmic contents, including DNA components into the microenvironment [1]. In addition, many living bacteria such as *Acinetobacter*, *Azotobacter*, *Bacillus*, *Deinococcus*, *Neisseria* and *Pseudomonas* release DNA into the surrounding environment during cell growth [1]–[6]. These bacteria benefit in several ways from free extracellular DNA and its degradation product [7]–[11]. For instance, the uptake of extracellular DNA, from the same or different organisms, promotes the evolution of bacteria. This occurs via horizontal gene transfer, such as transformation, transduction, or conjugation between bacteria [7], [8]. Extracellular DNA is also required for the initial establishment of bacterial biofilms, such as in *Pseudomonas aeruginosa*. The degradation of extracellular DNA by DNase I can strongly inhibit biofilm formation [9], [10]. Extracellular DNA, both homospecific and heterospecific, is known as an important nutrient source for organisms [11]. However, if extracellular DNA is not degraded immediately, it can threaten the survival of organisms by reincorporating damaged bases into the genome [12]. In most case, extracellular DNA components are degraded by extracellular nucleases secreted by many kinds of bacteria [13]–[16]. As a result, a threat to the organism is removed, and its by-products, dNMPs, are a nutrient source for bacteria [11], [12], [16].

*Deinococcus radiodurans* are extremely resistant to ionizing radiation, UV radiation, hydrogen peroxide and desiccation [12], [17]–[21]. The high resistance of this bacterium to reactive oxygen species (ROS) results from the strong ability of oxidative resistance [22] and an efficient DNA repair mechanism [23], [24]. Ionizing radiation [17] or UV [25] radiation attacks intracellular DNA producing large amounts of damaged oligonucleotides within the nucleotide pool [26]. These damaged oligos are exported into the surrounding medium and finally degraded [27]. However, it is not known if *D. radiodurans*, the most ionizing radiation resistant bacteria, has the ability to reuse these damaged extracellular DNA fragments.

Recently, Daly *et al.* demonstrated that *D. radiodurans* ultrafiltrate, which was enriched in Mn, phosphate, peptides, nucleosides and bases, could protect proteins from ionizing radiation-induced ROS damage [28], [29]. These findings implied that degradation and re-absorption of damaged DNA components might contribute to this organism's extreme ROS resistance. Here, we investigated the effects of extracellular DNA fragments and dNMPs on cell growth, H_2_O_2_ resistance, as well as UV and gamma-radiation in both *D. radiodurans* and *E. coli*. Our results indicated that the uptake of extracellular DNA fragments represented a new mechanism of protection from oxidative damage.

## Results

### Extracellular DNA fragments inhibit the growth of *D. radiodurans* but not *E. coli*

**Figure 1:**
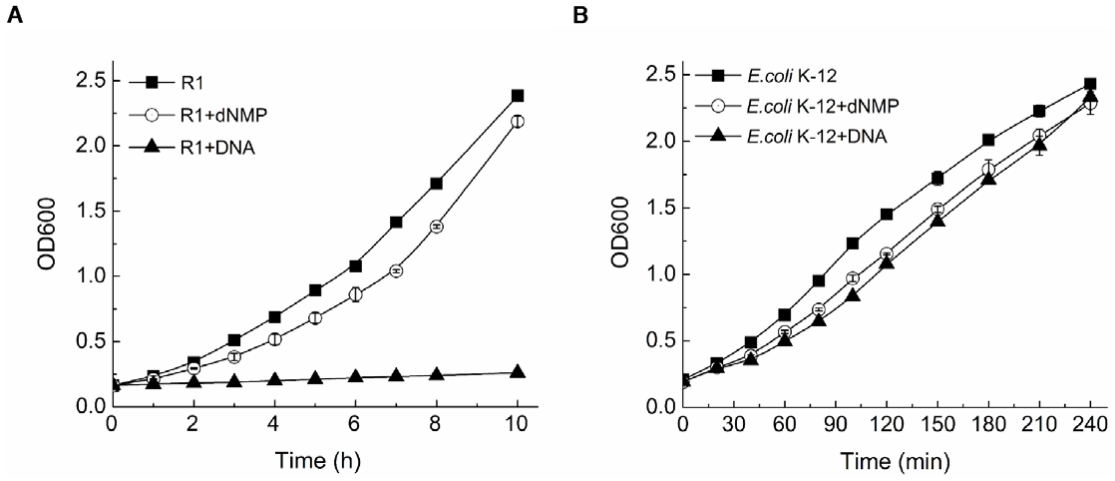
*D. radiodurans* and *E. coli* cell growth after DNA fragments or dNMPs treatment. (A) Growth of *D. radiodurans* after the addition of 3.6 mg/ml DNA fragments or dNMPs. (B) Growth of *E. coli* K-12 after the addition of 3.6 mg/ml DNA fragments or dNMPs. Values are the mean ± standard deviation of three independent experiments. R1, *D. radiodurans* wild type strain.

Free extracellular DNA is abundant in the environment, and its existence may have an effect on the growth of bacteria. To understand this effect, we grew *D. radiodurans* and *E. coli* cells in the presence or absence of 3.6 mg/ml DNA fragments or dNMPs. We observed large amounts of DNA fragments resulted in a distinct growth inhibition of *D. radiodurans*. Absorption reading (OD_600_) was 0.26 after 10 hours in the presence of DNA fragments, while control cells OD_600_ reading was 2.38 and that with dNMPs treatment was 2.19 (Fig. 1A). In *E. coli*, the OD_600_ values of control, DNA fragments and dNMPs treated groups were almost the same (OD_600_≈2.3) after 4 hours, which indicates neither DNA fragments nor dNMPs affect the growth of *E. coli* (Fig. 1B). These data suggested that DNA fragments and dNMPs have different effects on *D. radiodurans* and *E. coli* growth rates. Extracellular DNA fragments instead of dNMPs were harmful to *D. radiodurans* cells growth.

### Extracellular dGMP greatly enhances *D. radiodurans* tolerance to H_2_O_2_

**Figure 2:**
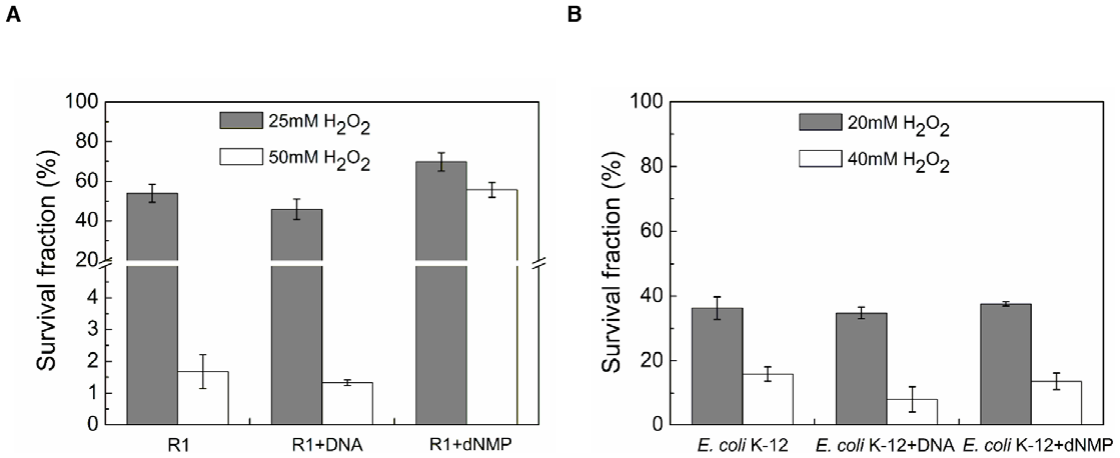
H_2_O_2_ sensitivity in *D. radiodurans* and *E. coli* treated with DNA fragments or dNMPs. (A) Sensitivity of *D. radiodurans* to H_2_O_2_ after the addition of 3.6 mg/ml DNA fragments or dNMPs. (B) Sensitivity of *E. coli* K-12 to H_2_O_2_ after the addition of 3.6 mg/ml DNA fragments or dNMPs. Each data point represents the mean±SD of three replicates. R1, *D. radiodurans* wild type strain.

**Figure 3:**
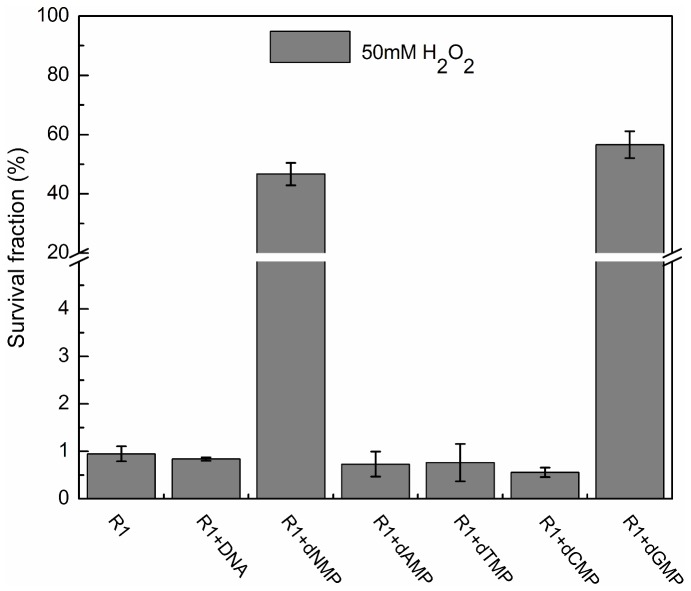
H_2_O_2_ sensitivity in *D. radiodurans* treated with dAMP, dTMP, dCMP or dGMP. Sensitivity of *D. radiodurans* to 50 mM H_2_O_2_ with the addition of DNA fragments (3.6 mg/ml), dNMPs, dAMP, dTMP, dCMP or dGMP (10 mM for each). Each data point represents the mean±SD of three replicates. R1, *D. radiodurans* wild type strain.

The influence of extracellular DNA fragments or dNMPs on *D. radiodurans* and *E. coli* cell survival under oxidative stress was evaluated. The presence of DNA fragments caused a modest decrease in H_2_O_2_ resistance in *D. radiodurans* (Fig. 2A). Similarly, the presence of DNA fragments did not have an obvious effect on *E. coli* resistance to H_2_O_2_ as well (Fig. 2B). However, the survival rate of *D. radiodurans* was dramatically increased when 10 mM dNMPs was present (Fig. 2A). There was a 33-fold increase in survival when compared to samples without dNMPs treatment. In *E. coli*, no distinct difference was observed between either the DNA fragments or dNMPs treatment groups (Fig. 2B). To understand which dNMPs accounted for this effect, dAMP, dTMP, dCMP and dGMP were separately added to *D. radiodurans* growths. Here we observed only dGMP had an effect, which dramatically increased H_2_O_2_ resistance by approximately 57-fold (Fig. 3). In addition, dGMP enhanced the resistance to gamma-radiation, but not UV (Fig. S1A). In sum, extracellular dGMP has an important role in *D. radiodurans* anti-oxidation, but not in *E. coli*.

### Extracellular dGMP induces KatA activity

**Figure 4:**
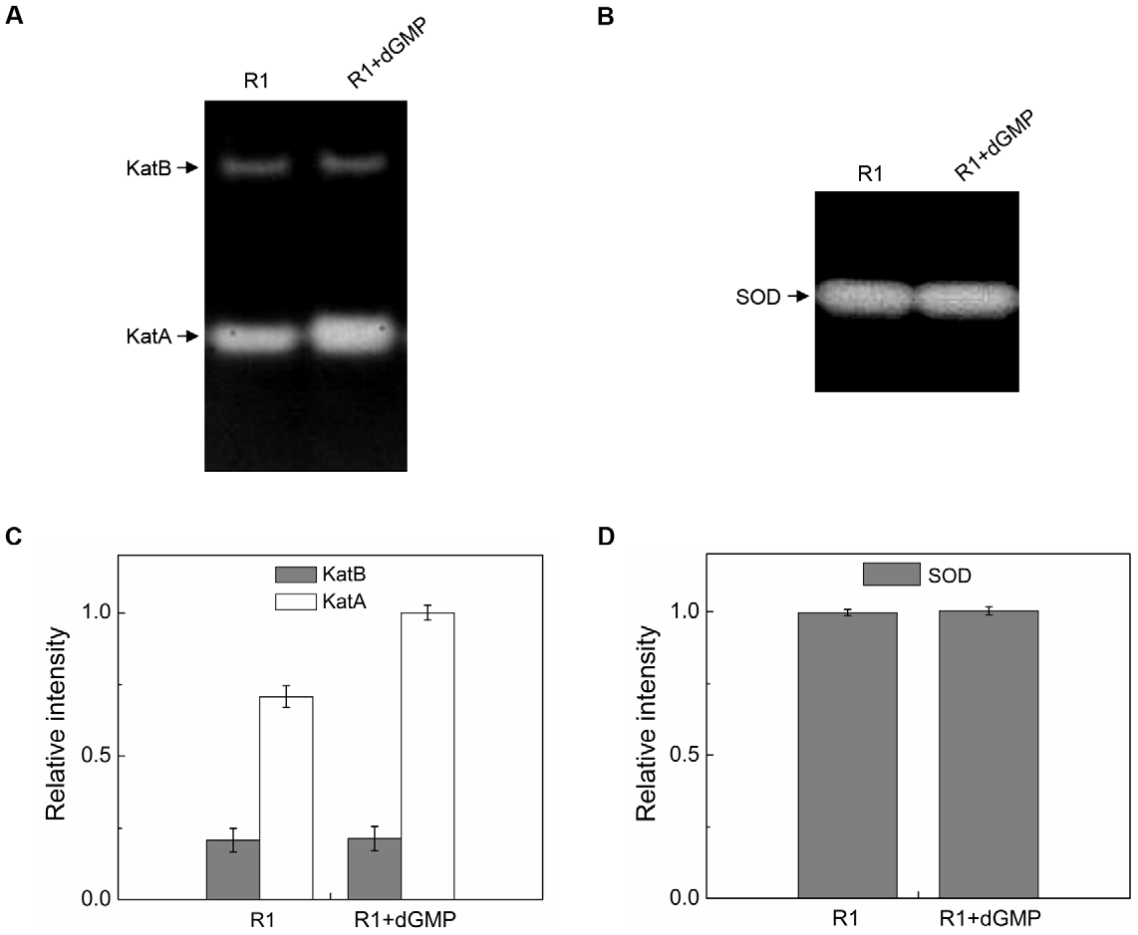
Addition of extracellular dGMP increases the activity of KatA in *D. radiodurans*. (A) Extracellular dGMP (2.5 mM) increased the activity of KatA, but not KatB. (B) Extracellular dGMP (2.5 mM) had no effect on the activity of SOD. (C) and (D) Quantification of the intensity of bands was performed using ImageJ. Each sample contains 80 µg of total protein. Values are the mean ± standard deviation of three independent measurements. R1, *D. radiodurans* wild type strain; KatA, catalase A; KatB, catalase B.

In *D. radiodura*ns, catalases and SODs protect proteins from ROS-mediated damage *in vivo* [30]. PAGE activity-staining assay reveals that *D. radiodurans* stain possesses activity corresponding to two catalases and one SOD, and stains that carry mutations in these genes (*katA and sodA*) are more sensitive to ionizing radiation than wild type [30]. In order to understand how dGMP increases *D. radiodurans* tolerance to H_2_O_2_, the activity change in catalases and SODs was measured after 2.5 mM dGMP was added to growth medium by PAGE activity-staining assay. The additional dGMP enhanced whole cell KatA activity by ∼50%, but had no effect on KatB or any of the SODs assayed (Fig. 4A/4B). It is possible that extracellular dGMP increases *D. radiodurans* tolerance to H_2_O_2_ by inducing KatA catalase activity *in vivo*.

### Extracellular dGMP stimulates transcription of anti-oxidation related genes

To better understand extracellular dGMP's involvement in anti-oxidation, the expression patterns of ROS response genes were investigated using Real-time quantitative PCR (Table 1). The addition of dGMP increased the transcriptional level of *katA* gene (*dr1998*) about 2.8-fold, but not any other catalase or SOD genes, which agrees with the results from the PAGE activity-staining assay. In other words, extracellular dGMP induces the activity of KatA by increasing the expression level of KatA *in vivo*.

**Table 1:** Influence of dGMP (2.5 mM) on *D. radiodurans* transcription levels.

ORF	Annotation	Fold	*p* value
**catalase and sod genes**			
DR1998	catalase	2.80	5.16E-06
DRA0146	catalase	1.01	0.85
DRA0259	catalase	0.65	2.12E-05
DR1279	Mn family superoxide dismutase	0.88	0.18
DR1546	Cu/Zn superoxide dismutase	0.95	0.29
DRA0202	Cu/Zn superoxide dismutase	0.73	0.78
**other genes**			
DR2283	manganese ABC transporter permease	2.98	1.79E-05
DR2523	Manganese/iron transport system substrate-binding protein	1.36	0.0003
DR2539	Mn-dependent transcriptional regulator	2.25	0.00014
DRB0016	iron complex transport system ATP-binding protein	2.79	0.0047
DRB0092	starvation-inducible DNA-binding protein	2.49	8.73E-05
DRB0121	iron ABC transporter, ATP-binding protein	1.62	1.70E-05
DRB0124	iron-chelator utilization protein, putative	9.71	2.43E-06
DRB0067	extracellular nuclease	2.00	0.0005
DR2244	sensory transduction histidine kinase	2.01	0.0032

The high intracellular Mn/Fe ratio in *D. radiodurans* could contribute to its remarkable resistance to environmental stresses [28], [29]. Here, the extracellular dGMP also increased the transcription levels of genes regulating the intracellular Mn/Fe ratio. DR2244, a sensory transduction histidine kinase, was induced by extracellular dGMP, which might be a signaling response to oxidative stress. Considering the role of extracellular nuclease in degrading extracellular DNA, the transcription of *drb0067*, the extracellular nuclease gene in *D. radiodurans*, was also investigated. When extracellular dGMP was present, transcription of *drb0067* was stimulated (2-fold), thus enhancing the degradation of extracellular DNA fragments and increasing the pool of dNMPs.

### *Drb0067* encodes the only extracellular nuclease in *D. radiodurans*

**Figure 5:**
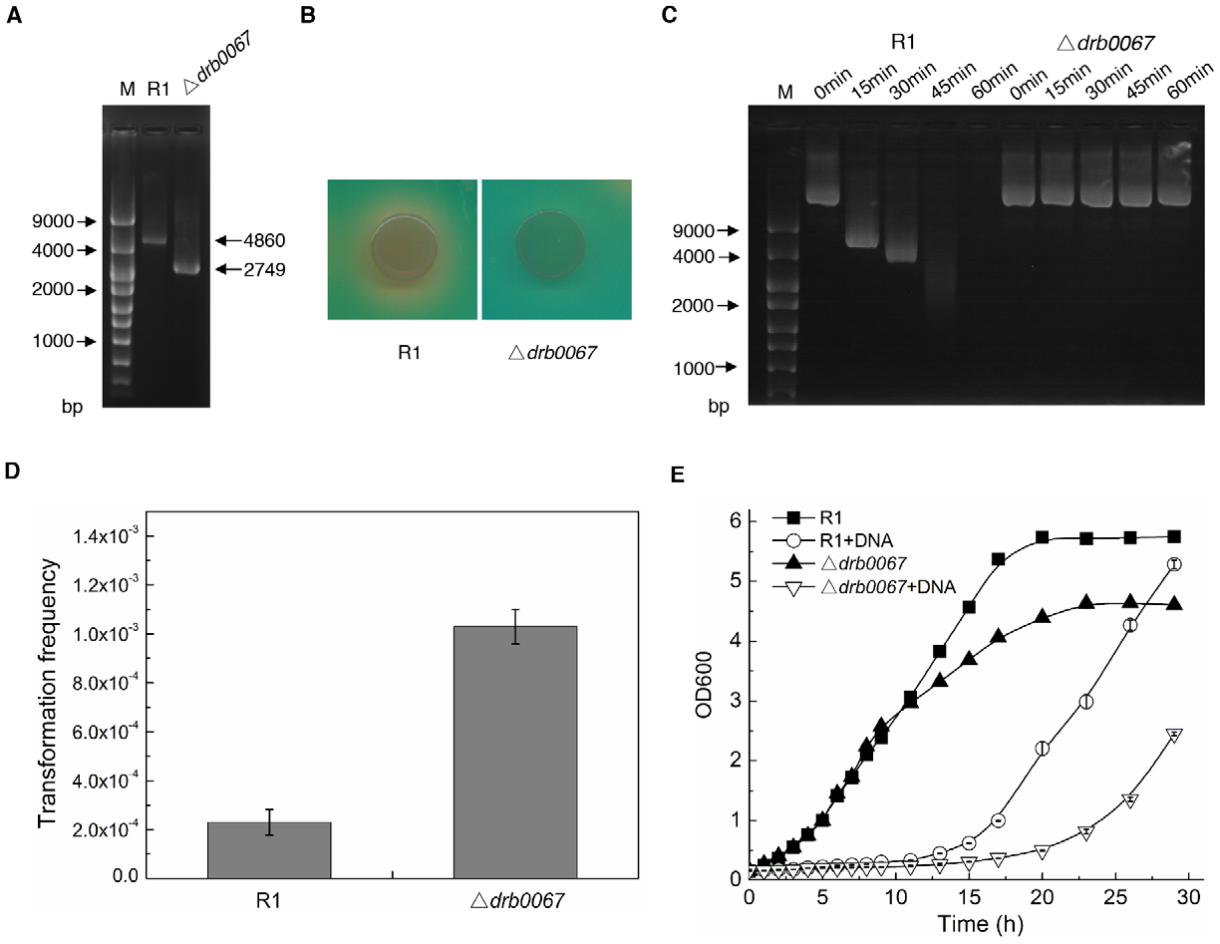
Disruption of *drb0067* gene and its phenotypes test. (A) PCR analysis of the mutant with primers 0067upF and 0067downR. (B) DNase Test Agar with Methyl Green to analyse R1 and Δ*drb0067*. 20 µl of cells (OD_600_≈1.0) were dripped onto DNase test agars plates. (C) Enzymatic activity to analyse R1 and Δ*drb0067*. The cells were cultured in TGY until the OD_600_≈2.5–3.0, and then centrifuged to collect the supernatant for enzyme reaction. (D) Transformation analysis of R1 and Δ*drb0067*. 1 µg pRADK plasmid was used for each transformation. (E) Growth of R1 and Δ*drb0067* with the addition of 3.6 mg/ml DNA fragments or dNMPs. M denotes molecular standards. All the experiments are performed three times and values are mean ± standard deviation. R1, *D. radiodurans* wild type strain; Δ*drb0067*, the *drb0067* null mutant.

Upon investigation of the *D. radiodurans* genome, we found that *drb0067* is the only extracellular nuclease gene. To further explore the role of DRB0067 in *D. radiodurans*, a *drb0067* null mutant (Δ*drb0067*) was constructed and verified by PCR (Fig. 5A). To test the mutant and wild type strains ability to degrade extracellular DNA, each strain was inoculated onto DNase test agars plates. A distinct clear zone was observed surrounding the wild type cells, but not Δ*drb0067* (Fig. 5B). Moreover, there was no nuclease activity detected in the culture medium of Δ*drb0067* (Fig. 5C). Bases on these two assays, we demonstrated DRB0067 is the only extracellular nuclease in *D. radiodurans*. And this extracellular nuclease is secreted through the secretory pathway since the deletion of *secD*/*secF* gene (*dr1822*) inhibited the secretion of DRB0067 (data not shown).

The extracellular nucleases act as a modulator for natural transformation in some bacteria, such as *Vibrio cholerae* [16]. Next, we investigated the transformation frequency for the Δ*drb0067* strain and found the transformation efficiency was ∼4.5-fold higher than the wild type strain (Fig. 5D). These results suggest that the DRB0067 protein is also an important modulator for natural transformation in *D. radiodurans*.

We next investigated the effect of DNA fragments on growth in the wild type and Δ*drb0067* strains. In the absence of extracellular DNA fragments, wild type and Δ*drb0067* strains had similar growth patterns except that the mutant strain (OD_600_≈4.61) exhibited slightly lower OD readings than wild type (OD_600_≈5.75) at the stationary phase. However, in the presence of DNA fragments, Δ*drb0067* grwoths were more sensitive than the wild type growths (Fig. 5E). Under these conditions, the wild type strain entered stationary phase after 29 hours (OD_600_≈4.60), whereas Δ*drb0067* was still in logarithmic phase (OD_600_≈2.45) at that time. Therefore, the presence of DNA fragments resulted in a severe cell growth decline in the Δ*drb0067* strain when compared to wild type.

### Deletion of *drb0067* impairs H_2_O_2_ resistance of *D. radiodurans*

**Figure 6:**
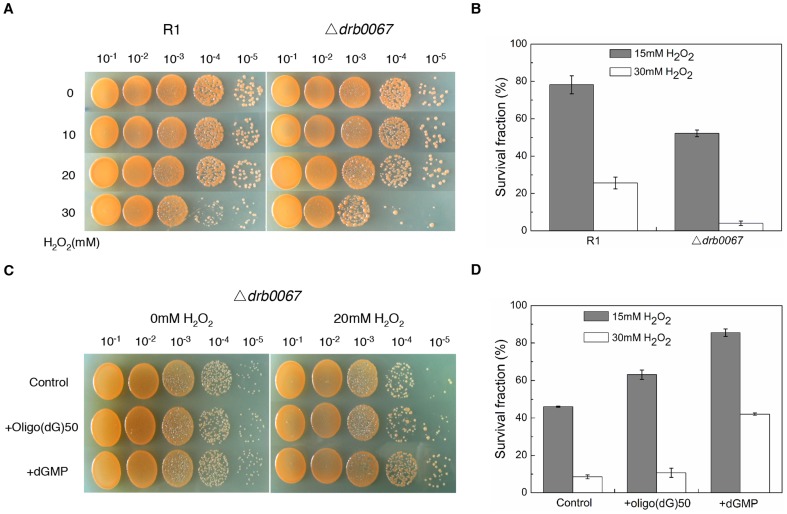
Sensitivity of Δ*drb0067* strain subjected to H_2_O_2_. (A) and (B) Sensitivity of R1 and Δ*drb0067* to different concentration of H_2_O_2_. (C) and (D) Sensitivity of Δ*drb0067* to different concentration of H_2_O_2_ with the addition of 0.05 mM oligo(dG)50 or 2.5 mM dGMP. Data represent the means ± deviations of three independent experiments. R1, *D. radiodurans* wild type strain; Δ*drb0067*, the *drb0067* null mutant.

Our experiments indicated that the extracellular dGMP, not extracellular DNA, enhanced the resistance of *D. radiodurans* to H_2_O_2_ and gamma-radiation. To understand the role of DRB0067 in this process, the H_2_O_2_ resistance of wild type and Δ*drb0067* strains were measured. These results, which were expected, revealed the *drb0067* mutation to have a decreased resistance to H_2_O_2_. The survival rate of the Δ*drb0067* strain was 6 times lower than that of the wild type strain under 30 mM H_2_O_2_ treatment (Fig. 6A/6B). Furthermore, the Δ*drb0067* was more sensitive to gamma-radiation, though not UV radiation (Fig. S1B). Next, the effects of oligo(dG)50 and dGMP on Δ*drb0067* resistance were investigated with varying concentrations of H_2_O_2_. Here, we found that the addition of dGMP (2.5 mM) restored the mutant strain's resistance to H_2_O_2_. The survival fraction with dGMP treatment was about 4 times higher than that with oligo(dG)50 treatment, and 5 times higher than control under the stress of 30 mM H_2_O_2_ (Fig. 6C/6D). In addition, *D. radiodurans* secreted more active extracellular nuclease after gamma-radiation treatment (Fig. S2). These results suggested that DRB0067 might be involved in ROS resistance through degradation of extracellular DNA to dNMPs, which increases the pool of dGMP. This pool then aids in enhancing the *D. radiodurans* tolerance to oxidative stress.

## Discussion

Here, we report extracellular dGMP enhanced the resistance of *D. radiodurans* to H_2_O_2_ and gamma-radiation. These findings suggest extracellular dGMP plays an important role in the organism's anti-oxidation pathway. Interestingly, we observed extracellular dGMP enhance the expression levels of KatA (DR1998) in *D. radiodurans*. These findings have yielded clues that may reveal the underlying mechanism of extracellular dGMP in anti-oxidation. Moreover, we found extracellular dGMP modulated expression of other genes, including one sensory transduction histidine kinase gene (*dr2244*), and genes involved in the regulation of manganese/iron. The up-regulation of these genes may enhance the tolerance of H_2_O_2_ and gamma-radiation as well.

Both cAMP and cGMP, as second messengers, have been widely studied in eukaryotes. It has been reported that cGMP can protect eukaryotic cells from oxidative stress, as in endothelial progenitors [31]. In bacteria, the production of cGMP has also been demonstrated. However, the physiological role of cGMP is still not well defined [32]. Recently Misra *et al.* reported that a DNA damage-induced signaling mechanism including secondary messengers and signaling enzymes exist in *D. radiodurans* [33]. Considering that the GC content (66.6%) in *D. radiodurans* is higher than most of other bacteria [27], we hypothesize that guanine base, obtained from the breakdown of extracellular dGMP [1], could be converted into cGMP after absorbed, and protect the cells from oxidative damage. However, further experiments are required to full characterize how dGMP enhances *D. radiodurans* tolerance to oxidative stress.

In *D. radiodurans*, DRB0067, encoded on the mega-plasmid, is an extracellular nuclease [27]. We demonstrated the absence of DRB0067 completely abolished nuclease activity from medium (Fig. 5B/5C). These results indicate DRB0067 is the only extracellular nuclease in this bacterium. Further experiments suggested this extracellular nuclease is secreted through the secretory pathway. Under normal growth conditions, DRB0067 is bound to the carotenoid-containing hexagonal layer [27]. Interestingly, the nuclease is released into the medium after ionizing radiation [34]–[36], indicating that this nuclease participates in *D. radiodurans* post-irradiation recovery. This hypothesis was indirectly supported by a transcriptome study reporting that the expression level of DRB0067 was induced after ionizing radiation [37]. Here, we have found direct evidence that deletion of *drb0067* decreases the survival ability of *D. radiodurans* after H_2_O_2_ or gamma-radiation treatment. Moreover, we report gamma-radiation enhances the secretion of DRB0067, indicating an important role for this protein in anti-oxidation. It is quite possible DRB0067 is required to degrade damaged DNA fragments that are exported after radiation damage to avoid genome mutagenesis. This mechanism also provides essential nutrition for cells' recovery. Interestingly, dGMP, one product from DNA degradation, dramatically stimulates *D. radiodurans* resistance to oxidative stress, which may indicate another purpose for DRB0067 induction after DNA damage stress.

Free extracellular DNA is a source of natural transformations. By degrading extracellular DNA, nucleases act as a modulator for natural transformation, such as the Dns protein in *V. cholerrae* [16]. In our experiments, an absence of DRB0067 increased the natural transformation rate of *D. radiodurans* (Fig. 5D), suggesting that DRB0067 acts as a natural transformation modulator in this bacterium. Free extracellular DNA is also a source of nutrients for organisms. However, the presence of large amounts of extracellular DNA inhibits *D. radiodurans* cell growth. Furthermore, the disruption of the *drb0067* gene amplified this effect. While in *E. coli* extracellular DNA had no effect on its growth. These findings support the importance of extracellular nuclease in this organism. Taken together, the degradation of extracellular DNA into dNMPs by extracellular nuclease DRB0067 serves many purposes in *D. radiodurans*. First by converting extracellular DNA into nutrients, thus reversing cell growth inhibition, and finally by enhancing *D. radiodurans* tolerance to oxidative stress.

## Materials and Methods

### Strains, media, and growth conditions

The bacterial strains and plasmids used in this study are listed in Table S1. *D. radiodurans* (ATCC 13939) was used as the wild-type strain and for construction of mutants. All cells were cultured at 30°C in TGY medium (0.5% Bacto tryptone, 0.3% Bacto yeast extract, 0.1% glucose) or on TGY plates containing 1.5% Bacto agar powder. *E. coli* strain DH5α was used for propagation of plasmids and was grown at 37°C on LB media with appropriate antibiotics.

### Disruption of the *drb0067* gene in *D. radiodurans*

Disruption of *D. radiodurans drb0067* gene was performed using the double crossover recombination method [38]. In brief, the 0067upF and 0067upR primers (Table S1) were used for the upstream fragment and 0067downF and 0067downR primers (Table S1) for the downstream fragment. The upstream and downstream were digested by *Hin*dIII and *Bam*HI respectively, and ligated to the *Bam*HI-*Hin*dIII fragment of the kanamycin resistance cassette containing the *gro*EL promoter. The kanamycin resistance cassette was obtained from pRADK, a shuttle plasmid modified from pRADZ3 [39]. The fragment was then transformed into *D. radiodurans* R1 with CaCl_2_ as described previously [40]. The mutant strain was obtained on TGY agar with 30 µg/ml kanamycin, and was confirmed by PCR with the primers 0067upF and 0067downR primers.

### Growth curve and survival fraction tests

Bacteria growth was determined using optical density data (OD) at 600 nm. The strains were cultured in 20 ml liquid TGY or LB medium until an OD_600_≈0.15 was reached, and DNA fragments (Herring sperm DNA from Sigma-Aldrich Company) or dNMPs, at a final concentration of 3.6 mg/ml, were added. The cultures were incubated with 250 rpm at 30°C or 37°C and samples were taken to measure the OD_600_ value at different time. All experiments were repeated in triplicate.

For the sensitivity assay, the strains were cultured in 5 ml liquid TGY medium until an OD of OD_600_≈0.75 was reached. Then DNA fragments, dNMPs, dGMP, or oligo(dG)50 were added to the growths (Table S1). Cultures were grown for another 3 hours. As a negative control autoclaved distilled water was added to a culture of each strain. After washed and diluted to an appropriate concentration with PBS solution, the cultures were treated with different concentrations of H_2_O_2_ (25 mM, 50 mM or 15 mM, 30 mM) for 30 min at 4°C. After treatment, the cells were plated on TGY plates and incubated at 30°C for 3 days before colonies were enumerated. The H_2_O_2_ survival assays on *E. coli* were performed as described above, except the cells were treated with 20 mM or 40 mM H_2_O_2_, plated on LB agar, and incubated at 37°C for 15 hours. Survival fraction (%) was calculated using the following equation: Survival fraction (%) = N_sample_/N_control_×100%, where N_control_ is the number of control colonies and N_sample_ is the number of H_2_O_2_ treated colonies. For the dripping test, the cultures were washed and serially diluted 1∶10 with PBS solution, and then treated with H_2_O_2_ (10 mM, 20 mM or 30 mM), gamma-radiation (2.5 h for 2 kGy) or UV (408 J/m^2^) separately [41]. 20 µl of cells were dripped onto TGY plates.

### Transformation test

The plasmid pRADK was used to test the effect of the extracellular nuclease DRB0067 on the natural transformation. Here, 1 µg plasmid was used for each transformation. The pRADK was then transformed into *D. radiodurans* R1 with CaCl_2_ as described previously [40]. Natural transformation frequencies were determined using the following equation: Natural transformation frequencies = N_TK_/N_TGY_, where N_TK_ is the number of clones on the TGY plates with 30 µg/ml kanamycin and N_TGY_ is the number of clones on the TGY plates.

### Extracellular nuclease activity assay

DNase test agars were used to test the extracellular nuclease activity. The cells were cultured in TGY until an OD of OD≈1.0 was reached, then 20 µl of cells were dripped onto DNase test agars plates (plates contain 42.0 g of DNase Agar Base (Qingdao Hope Bio-Technogy Co., Ltd, China), 0.05 g of methyl green and 2 g of glucose per liter of distilled water). Cultures were incubated at 30°C for 3 days. We used pRADK plasmid to test the activity of nuclease outside of the cells. The cells were cultured in TGY, and then centrifuged to collect the supernatant for enzyme reaction, during which 10 mM MgCl_2_ was added.

### Activity measurement of Catalase and SOD

Cells were treated with dGMP (2.5 mM) when they reached OD≈0.8, incubated for 3 hours and then disrupted with an ultrasonicator. The protein concentration of the supernatant was measured by the Bradford's method [42]. The catalase activity was assayed by the horseradish peroxidase-diaminobenzidine method [43]. In detail, samples were separated, using electrophoresis, in an 8% non-denaturing polyacrylamide gel matrix at 4°C for 4–5 hours (15 mA). The gel matrix was then washed with distilled water for 3 times. Next, it was incubated with a 0.06% H_2_O_2_ solution for 20 min under slow shaking. The gel was washed again and then incubated with FeCl_3_ (2%)/K_3_Fe(CN)_6_ (2%) (V/V = 1∶2) until bright strips appeared on the gel. For the assay of SOD activities, a 10% non-denaturing polyacrylamide gel was used according to the nitroblue tetrazolium-riboflavin method [44]. This method was nearly the same to the one described above except for the gel-running time (2–2.5 h) and the staining solution (2.45 mM nitroblue tetrazolium chloride (NBT), 28 mM TEMED, 28 µM riboflavin, and 100 mM EDTA, pH = 7.8). The gel was stained for 30 min and then exposed under lamplight until the bright strips appeared. For the activity measurement experiments 80 µg proteins were used per lane.

### Real-time quantitative PCR

Real-time quantitative PCR was used to determine the influence of the extracellular dGMP on the expression levels of Catalases, SODs and other genes of interest in *D. radiodurans*. In short, cells were grown to an OD_600_≈0.2 and then 2.5 mM dGMP was added. Cells were harvested by centrifugation at 4000 rpm for 5 min at 4°C when an OD_600_ of 0.4∼0.45 was reached. The extraction of total RNA and cDNA synthesis were performed as described previously [45]. SYBR *Premix Ex Taq*™ (TaKaRa Biotechnology (Dalian) Co, Ltd, China) was used for amplification, and all assays were performed using the STRATAGENE Mx3005*P*™ Real-time detection system.

## Supporting Information

Figure S1 **UV and gamma-ray sensitivity in R1 and Δ** ***drb0067*** **strains.** (A) Sensitivity of R1 to UV (408 J/m^2^) and gamma-radiation (2 kGy) with the addition of 10 mM dNMPs or 10 mM dGMP. (B) Sensitivity of R1 and Δ*drb0067* to UV (408 J/m^2^) and gamma-radiation (2 kGy). R1, *D. radiodurans* wild type strain; Δ*drb0067*, the *drb0067* null mutant.
(TIF)

Figure S2 **Gamma-radiation enhances the secretion of active extracellular nuclease.** The cells were cultured in TGY until the OD_600_≈1.0, treated with 2 kGy or 4 kGy gamma-radiation, and then centrifuged to collect the supernatant for enzyme reaction. The extracellular nuclease is secreted more from *D. radiodurans* after treatment. But no obvious extracellular nuclease activity change is observed from Δ*drb0067* after gamma-radiation treatment. M denotes molecular standards. R1, *D. radiodurans* wild type strain; Δ*drb0067*, the *drb0067* null mutant.
(TIF)

Table S1 **Strains, plasmids and primers used in this study.**
(DOC)
