# Supplementary figures and images for: Extracellular dGMP Enhances Deinococcus radiodurans Tolerance to Oxidative Stress
Source: PLoS One. 2013 Jan 24;8(1):e54420. doi: 10.1371/journal.pone.0054420 (PMC3554781; doi:10.1371/journal.pone.0054420)

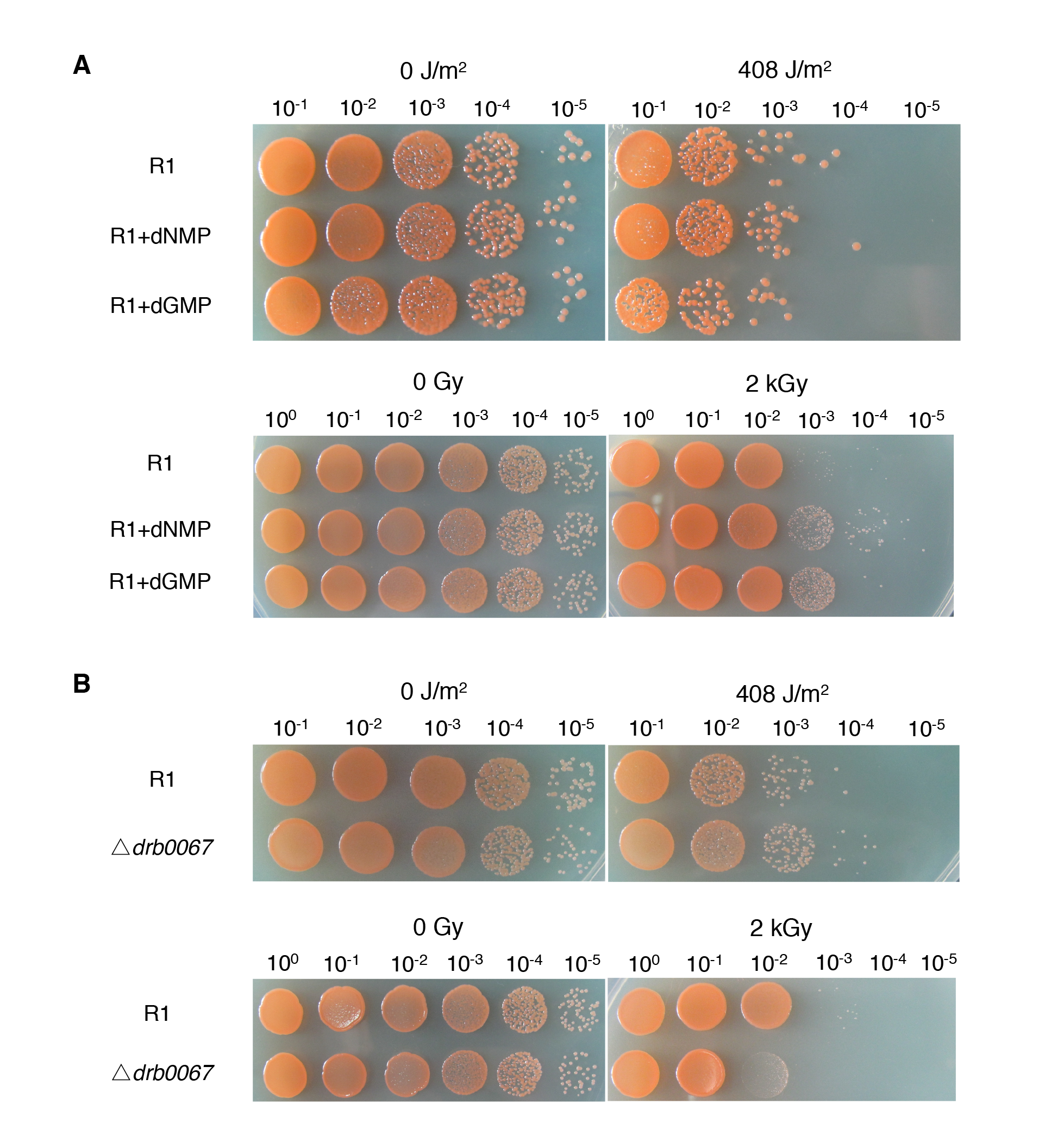

Supplement: Figure S1 — UV and gamma-ray sensitivity in R1 and Δ drb0067 strains. (A) Sensitivity of R1 to UV (408 J/m2) and gamma-radiation (2 kGy) with the addition of 10 mM dNMPs or 10 mM dGMP. (B) Sensitivity of R1 and Δdrb0067 to UV (408 J/m2) and gamma-radiation (2 kGy). R1, D. radiodurans wild type strain; Δdrb0067, the drb0067 null mutant. (TIF) [file pone.0054420.s001.tif]

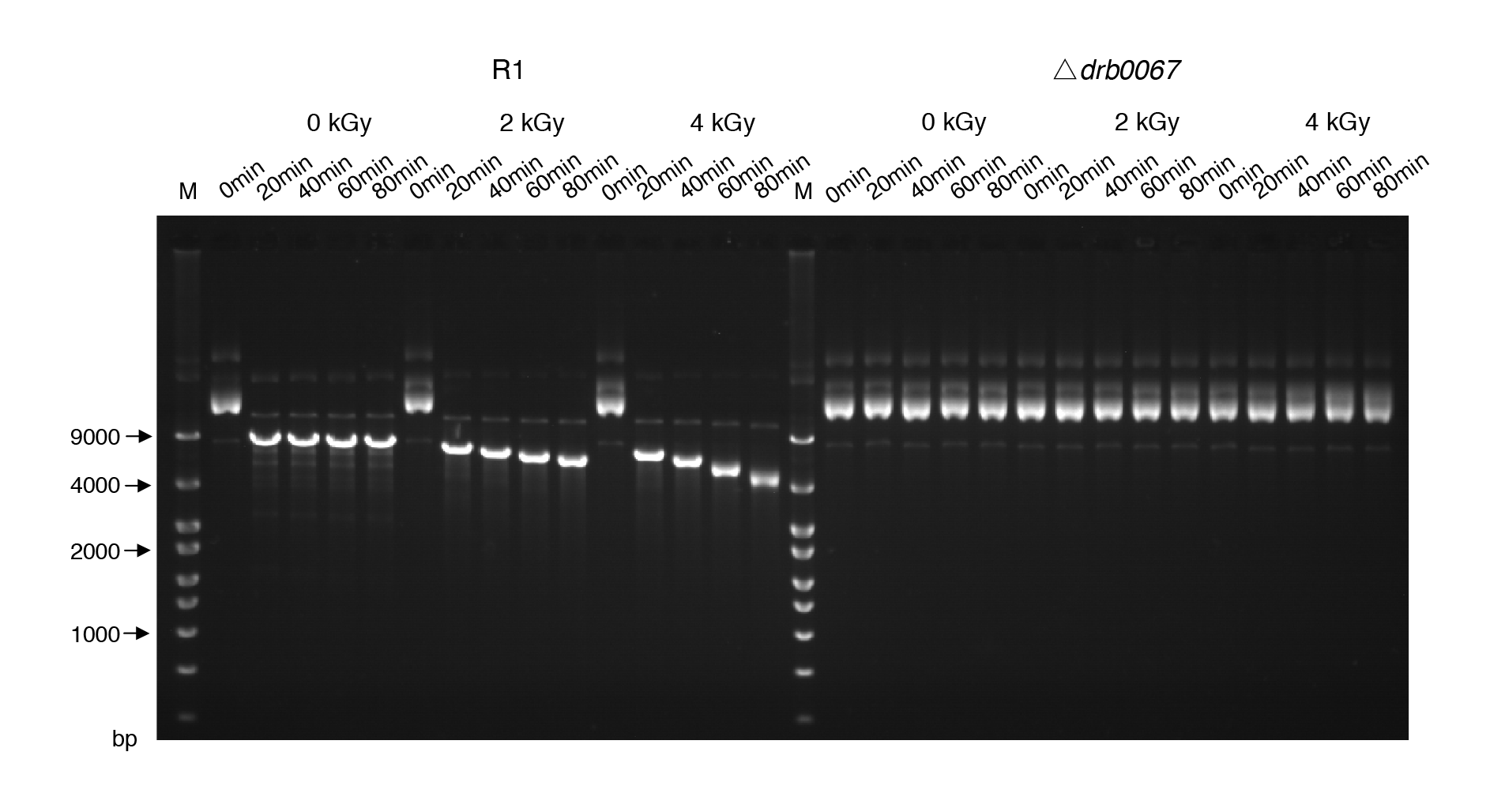

Supplement: Figure S2 — Gamma-radiation enhances the secretion of active extracellular nuclease. The cells were cultured in TGY until the OD600≈1.0, treated with 2 kGy or 4 kGy gamma-radiation, and then centrifuged to collect the supernatant for enzyme reaction. The extracellular nuclease is secreted more from D. radiodurans after treatment. But no obvious extracellular nuclease activity change is observed from Δdrb0067 after gamma-radiation treatment. M denotes molecular standards. R1, D. radiodurans wild type strain; Δdrb0067, the drb0067 null mutant. (TIF) [file pone.0054420.s002.tif]
