# Supplementary material for: Extracellular dGMP Enhances Deinococcus radiodurans Tolerance to Oxidative Stress
Source: PLoS One. 2013 Jan 24;8(1):e54420. doi: 10.1371/journal.pone.0054420 (PMC3554781; doi:10.1371/journal.pone.0054420)
Supplement: Table S1 — Strains, plasmids and primers used in this study. (DOC) [file pone.0054420.s003.doc]

| **Strains** | **Relevant marker** | **Source** |
| --- | --- | --- |
| *E. coli* DH5α | Propagation for plasmid | Invitrogen |
| *E. coli* K-12 | E-23370 | Invitrogen |
| *D. radiodurans* R1 | ATCC13939 | This lab |
| Δ*drb0067* | As R1, but *drb0067*::*kan* | This study |
| **Plasmids** | **Relevant marker** | **Reference or source** |
| pMD18-T | TA cloning vector | Takara |
| pRADK | *E. coli*-*D. radiodurans* shuttle vector |  |
| **Primers** | **Sequence (5′ → 3′)** | |
| 0067upF | CGGGGGAGGGGAGCAAGACAG | |
| 0067upR | AAGCTTTGCTGTTACCACCAGTAGGAC | |
| 0067downF | GGATCCAAACCAAAGTTGAGCTTGAAGG | |
| 0067downR | AGGCTGGGGAGAAGTGTTGC | |
| Oligo(dG)50 | GGGGGGAGGGGGGAGGGGGGAGGGGGGAGGGGGGAGGGGGGAGGGGGGAGGGGGGAGG | |

1. Meima R, Lidstrom ME (2000) Characterization of the minimal replicon of a cryptic Deinococcus radiodurans SARK plasmid and development of versatile Escherichia coli-D-radiodurans shuttle vectors. Applied and Environmental Microbiology 66: 3856-3867.
